# Features on Endoscopy and MRI after Treatment with Contact X-ray Brachytherapy for Rectal Cancer: Explorative Results

**DOI:** 10.3390/cancers14225565

**Published:** 2022-11-13

**Authors:** Petra A. Custers, Monique Maas, Doenja M. J. Lambregts, Regina G. H. Beets-Tan, Geerard L. Beets, Femke P. Peters, Corrie A. M. Marijnen, Monique E. van Leerdam, Inge L. Huibregtse, Baukelien van Triest

**Affiliations:** 1 Department of Surgery, Netherlands Cancer Institute-Antoni van Leeuwenhoek, P.O. Box 90203, 1006 BE Amsterdam, The Netherlands; 2 Department of Radiation Oncology, Netherlands Cancer Institute-Antoni van Leeuwenhoek, P.O. Box 90203, 1006 BE Amsterdam, The Netherlands; 3 GROW School for Oncology and Developmental Biology, Maastricht University, P.O. Box 616, 6200 MD Maastricht, The Netherlands; 4 Department of Radiology, Netherlands Cancer Institute-Antoni van Leeuwenhoek, P.O. Box 90203, 1006 BE Amsterdam, The Netherlands; 5 Department of Radiation Oncology, Leiden University Medical Centre, P.O. Box 9600, 2300 RC Leiden, The Netherlands; 6 Department of Gastroenterology, Netherlands Cancer Institute-Antoni van Leeuwenhoek, P.O. Box 90203, 1006 BE Amsterdam, The Netherlands; 7 Department of Gastroenterology and Hepatology, Leiden University Medical Centre, P.O. Box 9600, 2300 RC Leiden, The Netherlands

**Keywords:** rectal cancer, organ preservation, contact X-ray brachytherapy, imaging, MRI, endoscopy

## Abstract

**Simple Summary:**

Contact X-ray brachytherapy (CXB) after neoadjuvant (chemo)radiotherapy for rectal cancer is applied in selected patients aiming at organ preservation. However, limited data exist on features observed on endoscopy and MRI after treatment with CXB. On endoscopy, features observed in most patients 6 months after CXB are a flat, white scar, indicative for a clinical complete response (cCR), or tumor mass. On MRI, features indicative for a residual tumor are a focal tumor signal on T2W-MRI and a mass-like high signal on DWI. Due to treatment-related features observed early in follow-up, an irregular ulcer on endoscopy and a diffuse “reactive” mucosal signal on DWI, the distinction between a cCR and a residual tumor generally can be made at 6 months of follow-up. These results can help clinicians to interpret imaging features following CXB, ultimately, to identify patients with a cCR for Watch-and-Wait and to identify patients with a residual tumor for subsequent total mesorectal excision.

**Abstract:**

After neoadjuvant (chemo)radiotherapy for rectal cancer, contact X-ray brachytherapy (CXB) can be applied aiming at organ preservation. This explorative study describes the early features on endoscopy and MRI after CXB. Patients treated with CXB following (chemo)radiotherapy and a follow-up of ≥12 months were selected. Endoscopy and MRI were performed every 3 months. Expert readers scored all the images according to structured reporting templates. Thirty-six patients were included, 15 of whom obtained a cCR. On endoscopy, the most frequently observed feature early in follow-up was an ulcer, regardless of whether patients developed a cCR. A flat, white scar and tumor mass were common at 6 months. Focal tumor signal on T2W-MRI and mass-like high signal on DWI were generally absent in patients with a cCR. An ulceration on T2W-MRI and “reactive” mucosal signal on DWI were observed early in follow-up regardless of the final tumor response. The distinction between a cCR and a residual tumor generally can be made at 6 months. Features associated with a residual tumor are tumor mass on endoscopy, focal tumor signal on T2W-MRI, and mass-like high signal on DWI. Early recognition of these features is necessary to identify patients who will not develop a cCR as early as possible.

## 1. Introduction

In the early 2000s, Watch-and-Wait emerged as a treatment option for rectal cancer patients with a clinical complete response (cCR) following neoadjuvant (chemo)radiotherapy [1,2] for patients with a (near-)complete response or a small residual lesion after neoadjuvant (chemo)radiotherapy. Thus, for patients who do not develop a cCR, the standard treatment remains subsequent total mesorectal excision (TME). However, in patients with a strong wish for organ preservation, an additional radiation boost can be applied with the aim to achieve a cCR and to avoid TME [3,4,5,6,7,8]. With intracavity irradiation by contact X-ray brachytherapy (CXB), high radiation doses up to 90 Gy can be given to the residual tumor with minimal impact on the surrounding tissue [9]. As a result, cCR rates between 64% and 86% and organ-preserving rates up to 57% have been reported in selected patients [4,5].

After conventional (chemo)radiotherapy, the response assessment consists of the combination of digital rectal examination, endoscopy, T2-weighted (T2W)-MRI, and diffusion-weighted MRI (DWI) [10]. A cCR has been defined as a flat, white scar on endoscopy, the absence of a residual tumor mass on T2W-MRI, and the absence of diffusion restriction on DWI [2,11]. The different features and the correlation with a residual tumor in this specific setting have been previously described in detail [11,12,13,14].

With a substantially higher dose per fraction, the features on endoscopy and MRI after CXB may well be different from those typically observed after (chemo)radiotherapy. Response assessment and follow-up features after additional CXB have not been well documented, with only a brief description in a case series of seven patients treated with a variety of treatment strategies [15]. DWI findings are not included in this case series and a systematic approach to describe the evaluation of features over time is lacking. More information on features observed on endoscopy and MRI after CXB is required for a better evaluation of the response and to allow an earlier identification of patients with a residual tumor that will never develop a cCR and who require TME.

The aim of this explorative study was to describe the early features observed on endoscopy and MRI and to correlate these features with the final tumor response of patients with rectal cancer treated with CXB after neoadjuvant (chemo)radiotherapy aiming at organ preservation.

## 2. Materials and Methods

Starting in December 2017, patients with rectal cancer treated with CXB at The Netherlands Cancer Institute are registered in a prospective trial registry approved by the local institutional review board. For the present study, all patients with biopsy-proven rectal cancer with the following inclusion criteria were selected from the prospective trial registry: (1) CXB was performed for a small residual lesion after neoadjuvant (chemo)radiotherapy to aim for organ preservation, (2) neoadjuvant (chemo)radiotherapy consisted of either short-course radiotherapy (5 × 5 Gy) or (chemo)radiotherapy (28 × 1.8 Gy or 25 × 2 Gy in combination with capecitabine) followed by a long waiting interval, and (3) patients had a minimum follow-up of 12 months or an earlier biopsy-proven tumor remnant. All patients in the prospective trial registry provided informed consent.

### 2.1. Contact X-ray Brachytherapy

CXB was performed in an outpatient setting. A phosphate enema was given before the start of the procedure. Patients were placed in the lithotomy position, and, prior to each fraction, the tumor was visualized by rigid endoscopy. The Papillon 50 machine (Ariane Medical Systems, Derbyshire, UK) delivered CXB using 50 kVp X-rays through the rectal treatment applicator (size 20 mm, 25 mm, 30 mm depending on the size of the residual lesion), which was placed directly on the residual tumor. All patients received three fractions with a surface dose of 30 Gy per fraction with a 2-week interval. A fourth fraction was optional in case of a bowel movement causing repositioning of the tumor outside the applicator during the treatment procedure.

### 2.2. Follow-Up

Follow-up was performed according to a standardized 5-year follow-up schedule, with digital rectal examination, flexible endoscopy, and MRI. In general, follow-up was performed every 3 months in the first 2 years and every 6 months thereafter. Follow-up to detect distant metastases, including CT scans and carcinoembryonic antigen (CEA) levels, was performed according to the Dutch guidelines.

Endoscopy was performed with Olympus processor EVIS EXERA III with CF-190 high-definition endoscopes with an option for narrow band imaging (NBI). Pictures were taken from the (surrounding) area of the former tumor using white light endoscopy and NBI on indication. Biopsies were only taken in case of a suspected residual tumor.

MRI scans were performed at 1.5 T on an Intera Achieva MR system (Philips Medical Systems, Best, The Netherlands) using a phased array surface coil with the patients in a feet-first supine position. Fifteen minutes before the MRI scan was performed, a phosphate enema was given to reduce air artifacts. The standard MRI protocol included T2W fast spin echo sequences in three orthogonal directions (sagittal, transverse, and coronal) and a transverse DWI sequence with b1000 as the highest b-value. The transverse T2W and DWI sequences were angled in an identical plane perpendicular and parallel to the axis of the former rectal tumor. Apparent diffusion coefficient (ADC) maps were automatically generated by the operating system.

### 2.3. Evaluation of Endoscopy and MRI

The endoscopic images and MRI images were scored using structured scoring templates for the presence or absence of specific features, see reporting templates S1 and S2 of the Supplementary Material. All endoscopic images were scored by an expert reader (I.H.), and the presence of the most prominent feature was scored: (1) flat, white scar, (2) adenomatous tissue, (3) regular ulcer, (4) irregular ulcer, and (5) tumor mass.

**Figure 1:**
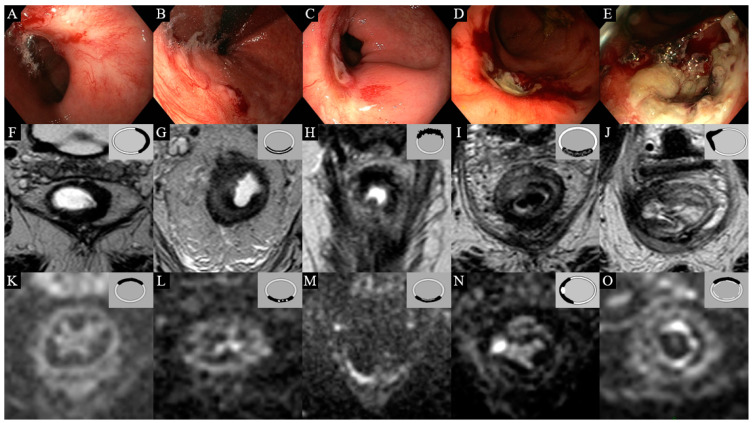
Features on endoscopy, T2W-MRI, and DWI. (**A**) Flat, white scar on endoscopy; (**B**) adenomatous tissue on endoscopy; (**C**) ulcer with regular borders on endoscopy; (**D**) ulcer with irregular borders on endoscopy; (**E**) tumor mass on endoscopy; (**F**) regular homogeneous fibrosis on T2W-MRI; (**G**) layered fibrosis on T2W-MRI; (**H**) irregular fibrosis on T2W-MRI; (**I**) heterogeneous fibrosis on T2W-MRI; (**J**) ulceration on T2W-MRI; (**K**) no high signal on DWI; (**L**) small spots of high signal on DWI; (**M**) linear high signal on DWI; (**N**) mass-like high signal on DWI; (**O**) a diffuse “reactive” mucosal diffusion signal on DWI.

MR images were evaluated by one of three expert readers (M.M., D.L., or R.B.T.). On T2W-MRI, the presence of an ulcer (yes/no), the morphology of the fibrosis (regular, layered, or irregular), and the signal of the fibrosis (homogeneous, heterogeneous, or focal tumor signal) were scored. On DWI, the presence and pattern of a focal high diffusion signal (none, small spots of high signal, linear high signal, and mass-like high signal) and the presence of a more diffuse “reactive” mucosal diffusion signal (present/absent) were scored. Figure 1 provides the features scored on endoscopy and MRI.

### 2.4. Standard of Reference

Patients were considered to have a residual tumor after treatment with CXB in the case of histopathological confirmation of carcinoma obtained by biopsies or surgery. Patients were considered to have a cCR when there was no sign of carcinoma on imaging or on histopathology during a minimum follow-up of 12 months.

### 2.5. Statistical Analysis

Statistical analyses were performed using IBM Statistical Package for the Social Sciences (SPSS version 27.0, Armonk, NY, USA). Descriptive statistics were used to report baseline characteristics (presented as percentages or medians with ranges) and features on endoscopy and MRI. Features observed at 3 months (±1 month) and 6 months (±1 month) of follow-up were described. Baseline characteristics and features were also separately reported for patients with a luminal cCR and patients with a residual tumor following CXB. In addition, diagnostic performance figures were calculated. Kaplan–Meier survival methods were used to analyze the organ-preservation rate, defined as the presence of an in situ rectum. Length of follow-up was calculated from the date of the last fraction until the date of the last follow-up moment or the date of TME surgery.

## 3. Results

For the present study, 36 patients treated with CXB following neoadjuvant (chemo)radiotherapy were included; 21 (58%) patients were male and the median age at diagnosis was 66 years (range 38–86). Baseline characteristics are provided in Table 1. The majority of patients were diagnosed with a cT3 tumor. (Chemo)radiotherapy was given to 29 patients, and short-course radiotherapy followed by a long waiting interval was given to 7 patients. The median size of the residual tumor prior to CXB on MRI and on endoscopy was 2.2 cm (range: 0.8–4.2) and 2.0 cm (range: 1.0–4.0), respectively. The median interval between (chemo)radiotherapy and the first fraction of CXB was 3 months (range: 2–38).

The median follow-up was 14 months (range: 2–43). Fifteen out of 36 patients had a luminal cCR following CXB; one of these patients had a luminal cCR but was treated with TME for a suspected mesorectal lymph node. Twenty-one patients had a histology-proven residual tumor following CXB; one of these patients was initially considered as cCR but had a luminal regrowth at 11 months and was therefore considered as a patient with a residual tumor. In total, 19 out of 36 patients were treated with TME following CXB after a median interval of 10 months (range: 5–24) (see Table 2). The corresponding 12-month organ-preservation rate was 62%. Three patients with a histology-proven residual tumor did not undergo TME, one patient due to comorbidity and two patients due to the presence of distant metastases. After the median follow-up of 14 months, distant metastases were present in five patients with residual disease and in two patients with a luminal cCR. In three of these patients, metastases were already present prior to treatment with contact X-ray brachytherapy.

### 3.1. Features on Endoscopy

**Figure 2:**
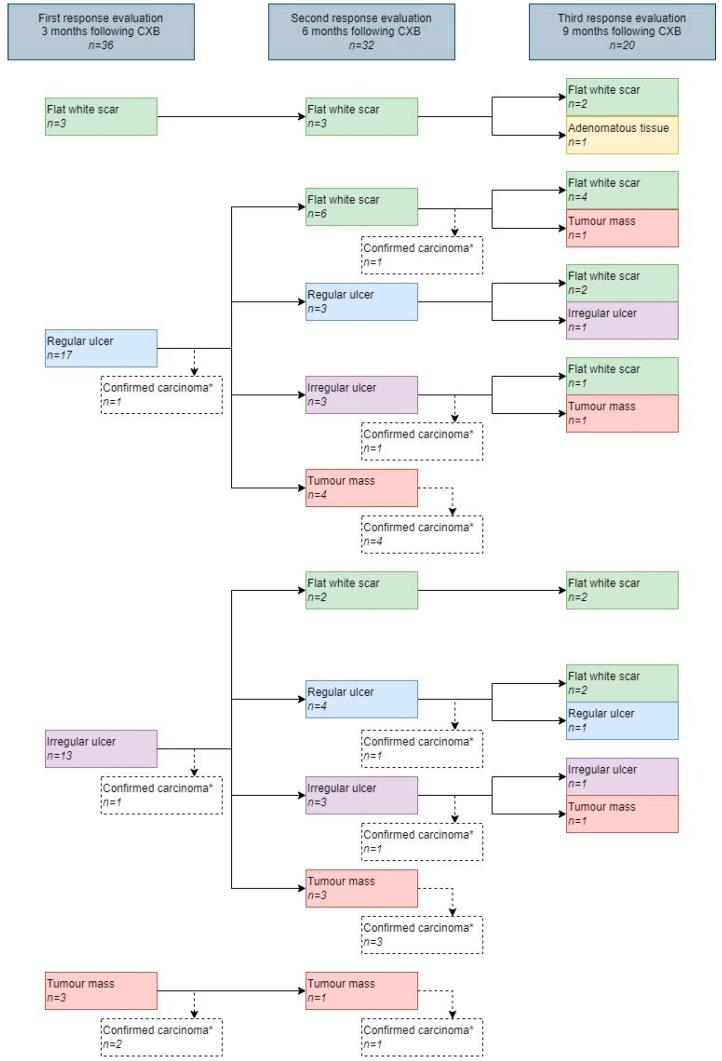
Features on endoscopy during follow-up. * No further follow-up on endoscopy and MRI of patients with histopathological confirmation of carcinoma obtained by biopsy or surgery.

**Figure 3:**
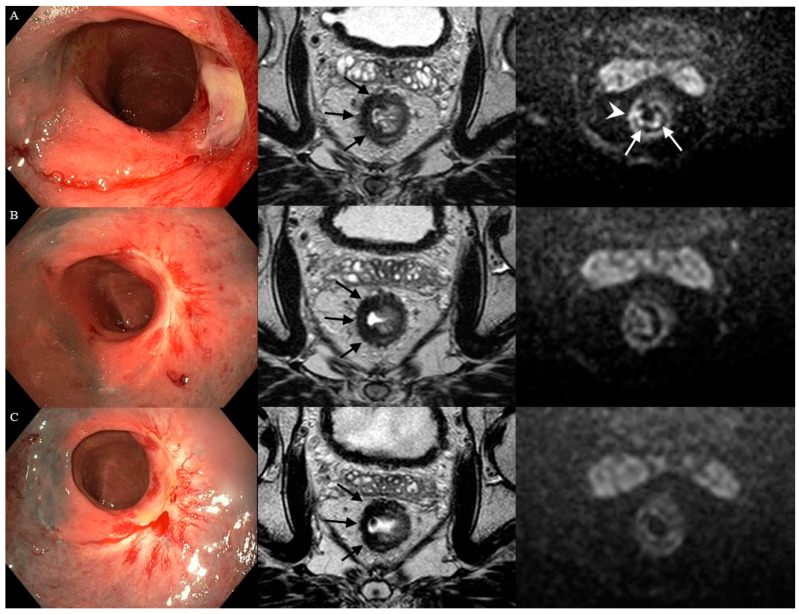
Case of a healing ulcer on endoscopy and the presence of a more diffuse “reactive” mucosal signal on DWI after contact X-ray brachytherapy. Three months following CXB (**A**), an irregular ulcer on endoscopy, irregular heterogeneous fibrosis (black arrows) on T2W-MRI, and small focal spots of high signal within the fibrosis (white arrowhead) in combination with a diffuse “reactive” mucosal signal on DWI were observed. Later, during follow-up at 7 (**B**) and 11 (**C**) months, the ulcer on endoscopy healed into a flat, white scar, the fibrosis on T2W-MRI became more regular and homogeneous, and, on DWI, the small focal spots and the diffuse “reactive” mucosal signal disappeared.

**Figure 4:**
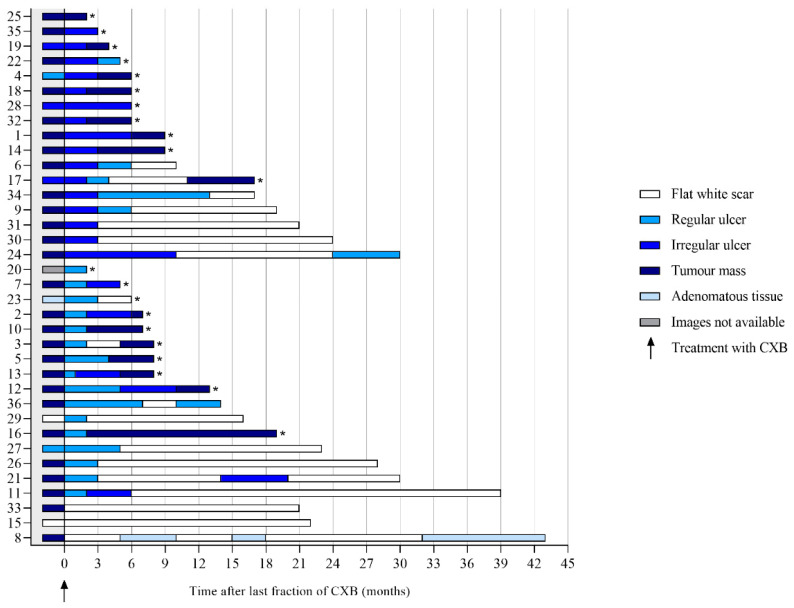
Features on endoscopy prior to and during follow-up after contact X-ray brachytherapy per patient. * Patients with histopathological confirmation of residual tumor.

Per patient, a median of three endoscopies (range 1–9) was performed, with an overall total of 144. The most frequently observed feature at 3 months was an ulcer (83%), which was described as regular in 57% (17/30) and irregular in 43% (13/30). A total of 27% of the ulcers healed into a flat, white scar at 6 months (see Figure 2). The most frequently observed feature at 6 months was a flat, white scar, observed in 34%, followed by tumor mass, which was observed in 25%. Of the patients with a flat, white scar, a regular ulcer, an irregular ulcer, or tumor mass at 3 months, 100%, 35%, 46%, and 0%, respectively, did achieve a cCR, whereas these figures at 6 months were 73%, 71%, 33%, and 0%. An example showing the follow-up of an irregular ulcer on endoscopy after CXB is provided in Figure 3. Features observed on endoscopy per patient are provided in Figure 4.

### 3.2. Features on T2W-MRI

Per patient, a median of three MRI scans (range: 0–9) was performed, with an overall total of 133. An ulceration was seen in 14% (5/35) at 3 months and in 28% (9/32) at 6 months. An ulceration was seen in both the patients who developed a cCR and the patients with a residual tumor. The morphology of fibrosis was predominantly irregular, in 40% (14/35) and 53% (17/32) at 3 and 6 months, respectively. Of the patients with irregular fibrosis at 3 and 6 months, 21% and 35% did develop a cCR. A layered aspect of the morphology of fibrosis was observed in 31% (11/36) at 3 months and 27% of those patients did develop a cCR. The signal of fibrosis was homogeneous in 40% (14/35) and heterogeneous in 60% (21/35) at 3 months. At 6 months, both aspects of the fibrosis were observed in about 40%. A focal tumor signal was observed in 16% (5/32) at 6 months; none of the patients with focal tumor signal developed a cCR. Features on T2W-MRI per patient are provided in Figures S1–S3 of the Supplementary Material.

### 3.3. Features on DWI

**Figure 5:**
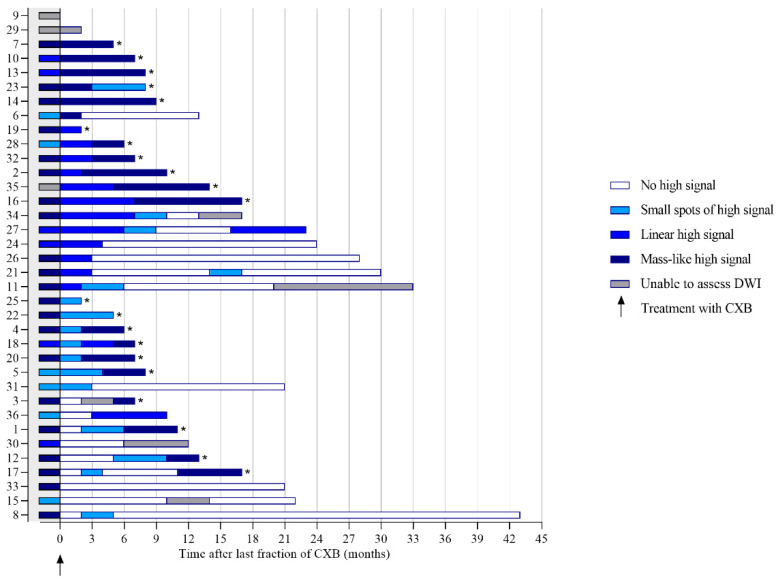
The morphology of the diffusion signal prior to and during follow-up after contact X-ray brachytherapy per patient. * Patients with histopathological confirmation of residual tumor.

**Figure 6:**
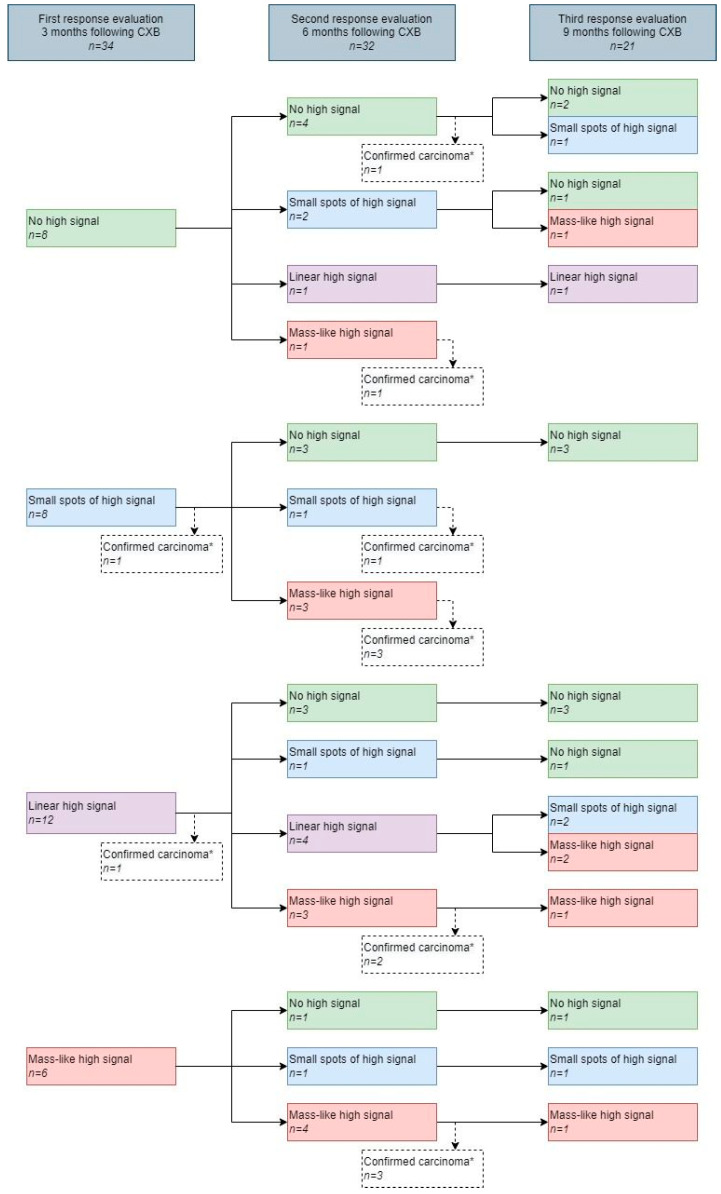
The morphology of the diffusion signal during follow-up. * No further follow-up on endoscopy and MRI of patients with histopathological confirmation of carcinoma obtained by biopsy or surgery.

The DWI scan was of sufficient quality in 34 and in 32 patients at 3 and 6 months, respectively. A diffuse “reactive” mucosal signal was present in 65% (22/34) at 3 months, which decreased to 41% (13/31) at 6 months, see Figure S4 of the Supplementary Material. An example of a diffuse “reactive” mucosal signal is shown in Figure 3. Morphology of diffusion signal on DWI at 3 months was predominantly linear in 35% (12/34) followed by a small spot of high signal and no high signal both in 24% (8/34). Of the patients with no high signal, small spots of high signal, a linear high signal, or mass-like high signal at 3 months, 62%, 12%, 50%, and 17% did proceed into a cCR. At 6 months, a mass-like high signal and no high signal, in 38% (12/32) and 31% (10/32), were most frequently observed (see Figure 5). Of the patients with no high signal or mass-like high signal, 80% and 0% did proceed into a cCR, respectively. Features on DWI per patient are provided in Figure 5. The morphology of the diffusion signal at 3, 6, and 9 months of follow-up is provided in Figure 6.

### 3.4. Diagnostic Performance of Endoscopy and MRI

Tumor mass on endoscopy, focal tumor signal on T2W-MRI, and mass-like high signal on DWI were seen at 3 months in 14%, 0%, and 24% of the patients with histopathological confirmation of carcinoma (see Table 3). At 6 months, these figures were 47%, 26%, and 58%, respectively. When combining endoscopy and DWI at 3 months, in 38% (8) of the patients with a residual tumor, either a tumor mass on endoscopy or a mass-like high signal on DWI was observed. When combining all three modalities at 6 months, in 68% (13) of the patients with a residual tumor, either a tumor mass on endoscopy, a focal tumor signal on T2W-MRI, or a mass-like high signal on DWI was observed.

## 4. Discussion

This explorative study describes the features observed on endoscopy and MRI in the first year after treatment with CXB following neoadjuvant (chemo)radiotherapy as an organ-preserving treatment strategy. In the first 3 months, the majority of the patients had ulcers on endoscopy that impress either regular or irregular, which do not indicate the presence or absence of any residual tumor. However, the incidence of ulcers decreased over time, whereas the incidence of flat, white scars and residual tumor masses increased. Consequently, the endoscopic distinction between a cCR and the patients with a residual tumor after CXB can more reliably be made at 6 months of follow-up. Features on T2W-MRI and DWI support this phenomenon, as a focal tumor signal on T2W-MRI, which was only observed in patients with a residual tumor, was first seen at 6 months of follow-up. On DWI, the majority of the patients at 3 months of follow-up had a linear high signal, a signal that cannot discriminate a cCR from a residual tumor. Nevertheless, at 6 months, a mass-like high signal was the most frequently observed feature, which did indicate the presence of a residual tumor. Features not indicative for either a cCR or a residual tumor were an ulceration on T2W-MRI and a diffuse “reactive” mucosal signal on DWI. However, these features were mainly observed in the first months of follow-up.

Based on our observations, features discriminating a cCR from a residual tumor were, in general, more frequently observed at 6 months of follow-up. Features indicative for a residual tumor after CXB were a tumor mass on endoscopy, a focal tumor signal on T2W-MRI, and a focal spot of high signal on DWI. A previously published study on poor responders following neoadjuvant (chemo)radiotherapy reported similar results [16]. For clinical practice, the early recognition of features associated with a poor response is of importance, as TME should be performed as early as possible in patients who will not develop a cCR, to ensure curative intent [4,5,17]. Features typically found in patients with a cCR following neoadjuvant (chemo)radiotherapy without CXB are a flat, white scar on endoscopy, homogeneous regular fibrosis on T2W-MRI, and the absence of a high signal on DWI [10,11,16]. As might be expected, these features were also observed in the present study and were present in more than half of the patients with a cCR at 6 months. Remarkably, a layered aspect of the fibrosis, a feature that was previously described as the “split scar” sign and was associated with a cCR after (chemo)radiotherapy, was observed in both the patients with a cCR and the patients with a residual tumor [18]. It may, therefore, be less useful in the setting of response monitoring after CXB.

On endoscopy, an irregular ulcer was observed in a substantial number of patients early during follow-up: at 3 months in 40% of the patients with a cCR. This high percentage is in contrast to a previous study on the predictive value of endoscopic features following (chemo)radiotherapy without CXB, in which an irregular ulcer was observed in only 1–7% of the patients with a cCR [14]. As ulcers on endoscopy are more frequently reported following high radiation doses given with intracavity irradiation [19], the presence of an irregular ulcer on endoscopy might be an early effect of the high boost dose itself. Another feature that is probably related to the effects of the CXB itself is the presence of a more diffuse “reactive” mucosal signal on DWI, which we consider to be a reactive inflammatory effect. This reactive signal was observed in over half of the patients at 3 months and decreased during follow-up. This reactive signal is probably similar to a previously reported high DWI signal observed during early follow-up after transanal endoscopic microsurgery (TEM), which was then reported as a potential cause for false positivity during early follow-up [20]. In line with the present study, this false-positive high signal was also reported to decrease over time in the post-TEM setting [20].

Due to the presence of the early reactive effects of CXB itself, the response after 3 months was often not unequivocal, reflected by the increased sensitivity and specificity of endoscopy and MRI over time. The first response evaluation at 3 months should, therefore, serve as a baseline, after which, at 6 months of follow-up, in general, a clear distinction between a cCR and a residual tumor can be made. In the present study, response assessment did include endoscopy, T2W-MRI, and DWI. The specificity to detect patients with residual disease at 6 months was high, but sensitivity was rather low. However, when combining the three modalities, over two-thirds of the patients with residual disease could be identified at 6 months. A multi-modality response assessment including endoscopy and MRI is therefore recommended by the authors. In addition, to monitor the evolution of the response during follow-up, the authors encourage the use of structured report templates to evaluate the endoscopy and MRI after CXB.

Of the 36 patients treated with CXB following neoadjuvant (chemo)radiotherapy in the present study, 21 patients (51%) did not develop a cCR and had a residual tumor following CXB. In light of the recently published data of the OPERA trial [21,22], reporting a 3-year organ-preserving rate of 81%, the oncological results of the present study are modest. This difference can partly be explained by the difference in selection criteria for CXB. In the present study, patients initially diagnosed with more advanced rectal tumors and with larger residual tumors were included, especially in the group of patients with a residual tumor after treatment with contact X-ray brachytherapy; although, both patient groups are too small for a statistical comparison. Additionally, patients with an interval of more than 3 months between neoadjuvant (chemo)radiotherapy and CXB were included. For radiobiological reasons, a shorter interval between neoadjuvant (chemo)radiotherapy and CXB is advisable [23]. However, higher complete response rates were observed with an increasing interval up to 16 weeks after neoadjuvant (chemo)radiotherapy [24,25,26]. As such, performing CXB according to the treatment schedule of the OPERA trial, irrespectively of the tumor response following a 2–3-week interval after neoadjuvant (chemo)radiotherapy, may result in overtreatment in a substantial number of patients who may continue on to a cCR following conventional (chemo)radiotherapy only [21]. Due to advancing insight and the results of the present study, the authors nowadays use more strict selection criteria for treatment with CXB aiming at organ preservation, where patients with a residual tumor of more than 3 cm and/or an interval of more than 3 months after (chemo)radiotherapy are generally no longer considered for CXB, in accordance with the ESTRO consensus recommendations for CXB [27].

This study does have some limitations. The first limitation is the small sample size; therefore, the diagnostic performance of endoscopy and MRI and the descriptive results should be interpreted as explorative. A second limitation is the heterogeneity of the patients selected for this study. An interpretation of the oncological outcome of this study should, therefore, be made with caution. The third limitation is the lack of data on the nodal response as this study focused on the luminal response following CXB. However, when determining whether or not there is a cCR, the nodal response should, of course, be included. Fourth, the endoscopy and MRI were scored by expert readers only; whether or not these results can be generalized into daily clinical practice should be explored in further research. In addition, the images were scored by only one expert; as a result, no data are available on interobserver variation. The possibility of interobserver variation should also be explored in further research using several expert and non-expert readers.

## 5. Conclusions

This explorative study describes the early features observed on endoscopy and MRI of rectal cancer patients treated with CXB following neoadjuvant (chemo)radiotherapy aiming at organ preservation. Early recognition of features indicative for a residual tumor is of importance for clinical practice as patients with a residual tumor should be referred for TME surgery as early as possible. Features associated with a residual tumor are a focal tumor signal on T2W-MRI and a focal spot of high signal on DWI. An irregular ulcer on endoscopy and a more diffuse “reactive” mucosal signal on DWI might partly result from the high doses given with CXB; the presence of these features early in follow-up (after 3 months) is, therefore, likely mainly treatment related and not predictive for the final tumor response. These treatment-related features can obscure the response evaluation after 3 months; we, therefore, advise that, in general, the distinction between a cCR and a residual tumor should be made based on the findings observed on endoscopy and MRI starting 6 months following CXB. These results can help clinicians to interpret the features on endoscopy and MRI after CXB for rectal cancer.

## Figures and Tables

**Table 1:** Patient characteristics.

Characteristics	Total Cohort (*n* = 36)		Patients with a cCR (*n* = 15)		Patients with Residual Tumor (*n* = 21)	
Median age (years) (range)	66	38–86	67	54–86	66	38–79
Sex (n, %)						
Male	21	58	9	60	12	57
Female	15	42	6	40	9	43
Clinical tumor stage (n, %)						
cT2	9	25	6	40	3	14
cT3	26	72	8	53	18	86
cT4	1	3	1	7	0	0
Clinical nodal stage (n, %)						
cN0	20	56	9	60	11	52
cN1	12	33	6	40	6	29
cN2	4	11	0	0	4	19
Neoadjuvant radiotherapy (n, %)						
Short-course radiotherapy	7	19	7	47	0	0
(Chemo)radiotherapy	29	81	8	53	21	100
Clinical nodal stage prior to CXB (n, %)						
ycN0	34	94	14	93	20	95
ycN1	2	6	1	7	1	4
Clinical distant metastasis prior to CXB (n, %)						
ycM0	33	92	14	93	19	90
ycM1	3	8	1	7	2	10
Median size tumor prior to CXB on MRI (cm) (range)	2.2	0.8–4.2	1.9	1.0–3.0	2.0	0.8–4.2
Median size tumor prior to CXB on endoscopy (cm) (range)	2.0	1.0–4.0	2.0	1.0–2.5	2.0	1.0–4.0
Median interval neoadjuvant radiotherapy and CXB (months) (range)	3	2–38	2	2–21	4	2–38
Patients treated with TME surgery following CXB (n, %)	19	53	1	7	18	86

Abbreviations: CXB = contact X-ray brachytherapy, cCR = clinical complete response.

**Table 2:** Characteristics of TME surgery.

Characteristics	Total Cohort (*n* = 19)		Patients with a cCR (*n* = 1)		Patients with Residual Tumor (*n* = 18)	
Median interval end CXB and TME surgery (months) (range)	10	5–24	24		9	5–20
Pathological tumor stage of patients treated with TME surgery following CXB (n, %)						
ypT0	1	5	1	100	0	0
ypT1	1	5	0	0	1	6
ypT2	6	32	0	0	6	33
ypT3	10	53	0	0	10	56
ypT4	1	5	0	0	1	6
Pathological nodal stage of patients treated with TME surgery following CXB (n, %)						
ypN0	16	84	0	0	16	89
ypN1	3	16	1	100	2	11

Abbreviations: TME = total mesorectal excision, CXB = contact X-ray brachytherapy, cCR = clinical complete response.

**Table 3:** Diagnostic performance of different features on endoscopy and MRI at 3 and 6 months after treatment with contact X-ray brachytherapy.

	**Tumor Response**		**Diagnostic Performance %**				
**Features at 3 Months**	**Residual Tumor**	**cCR**	**Sensitivity**	**Specificity**	**PPV**	**NPV**	**Accuracy**
Tumor mass on endoscopy							
+	3 (TP)	0 (FP)	14	100	100	45	50
-	18 (FN)	15 (TN)					
Focal tumor signal on T2W-MRI							
+	0 (TP)	0 (FP)	0	0	0	0	0
−	0 (FN)	0 (TN)					
Mass-like high signal on DWI							
+	5 (TP)	1 (FP)	24	93	83	47	53
−	16 (FN)	14 (TN)					
	**Tumor Response**		**Diagnostic Performance %**				
**Features at 6 Months**	**Residual Tumor**	**cCR**	**Sensitivity**	**Specificity**	**PPV**	**NPV**	**Accuracy**
Tumor mass on endoscopy							
+	8 (TP)	0 (FP)	47	100	100	63	72
−	9 (FN)	15 (TN)					
Focal tumor signal on T2W-MRI							
+	5 (TP)	0 (FP)	26	100	100	48	56
−	14 (FN)	13 (TN)					
Mass-like high signal on DWI							
+	11 (TP)	0 (FP)	58	100	100	62	75
−	8 (FN)	13 (TN)					

Abbreviations: Residual tumor = histopathological confirmation of carcinoma; cCR = clinical complete response; TP = true positive; TN = true negative; FP = false positive; FN = false negative; PPV = positive predictive value; NPV = negative predictive value.

## Data Availability

Study data are available for review upon reasonable request.

## Supplementary Materials

The following supporting information can be downloaded at https://www.mdpi.com/article/10.3390/cancers14225565/s1. Reporting template S1: Structured endoscopy scoring template: Response evaluation after contact X-ray brachytherapy; Reporting template S2: Standardized MRI report template: Response evaluation after contact X-ray brachytherapy; Figure S1: The presence of an ulceration on T2W-MRI during follow-up after contact X-ray brachytherapy (CXB) per patient; Figure S2: Morphology of the fibrosis on T2W-MRI during follow-up after contact X-ray brachytherapy (CXB) per patient; Figure S3: Signal of the fibrosis on T2W-MRI during follow-up after contact X-ray brachytherapy (CXB) per patient; Figure S4: The presence of a reactive signal on DWI during follow-up after contact X-ray brachytherapy (CXB) per patient.
