# Supplementary material for: Features on Endoscopy and MRI after Treatment with Contact X-ray Brachytherapy for Rectal Cancer: Explorative Results
Source: Cancers (Basel). 2022 Nov 13;14(22):5565. doi: 10.3390/cancers14225565 (PMC9688812; doi:10.3390/cancers14225565)
Supplement: Supplementary file 1 [file cancers-14-05565-s001.zip › cancers-1968586-supplementary.pdf]

Supplementary file

Reporting template S1. Structured endoscopy scoring template – Response evaluation after contact x-ray brachytherapy

Structured endoscopy scoring template

Response evaluation after contact x-ray brachytherapy

Digital rectal examination

Palpable lesion?	Yes / No
If yes:	Soft minor abnormalities / stiff wall / flat ulcer / ulcer with elevated edges / clear residual tumour
If yes:	Distance lesion to anal verge ….. cm

Endoscopy

Is there a flat scar?	Yes / No
Is there residual adenomatous tissue?	Yes / No
Is there a residual ulcer?	Yes / No
If yes:	Small (<1cm) / large (≥1cm)
If yes:	Regular edges / irregular edges
Is there elevated tumour?	Yes / No
Are there telangiectasia?	Yes / No
Is there proctitis / bleeding?	Yes / No
Is there stenosis?	Yes / No
Is there a healing tendency?	Yes / No / Not applicable

Distance lesion to anal verge	….. cm
Size of lesion	….. cm

Most prominent feature on endoscopy

Flat scar	Yes / No
Residual adenomatous tissue	Yes / No
Ulcer with regular edges	Yes / No
Ulcer with irregular edges	Yes / No
Residual tumour mass	Yes / No

Confidence level tumour response

1. Definitely complete response
2. Probably complete response
3. Maybe (in)complete response
4. Probably incomplete response
5. Definitely incomplete response

Reporting template S2. Standardized MRI report template – Response evaluation after contact x-ray brachytherapy

Standardized MRI report template

Response evaluation after contact x-ray brachytherapy

T2W-MRI

The presence of an ulceration ☐ No ☐ Yes

If yes: Width ….. cm

If yes: Depth ….. cm

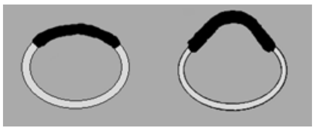

Morphology of the fibrosis ☐ Regular ☐ Layered ☐ Irregular

Maximal thickness of the fibrosis ….. cm

Circumference of the fibrosis ……….

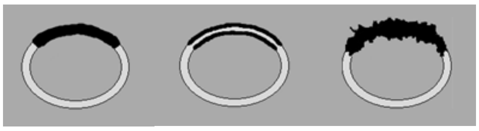

Signal of the fibrosis

☐ Homogeneous

☐ Heterogeneous

☐ Focal tumour signal

DWI-MRI

Reactive mucosal signal ☐ No ☐ Yes

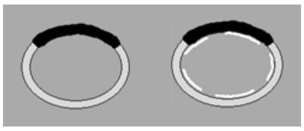

Morphology of the DWI signal

☐ No high signal

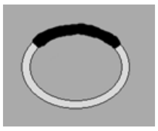

☐ Small spots of high signal

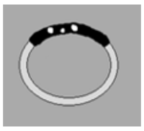

☐ Linear Signal

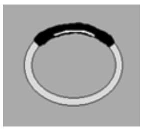

☐ Focal tumour signal

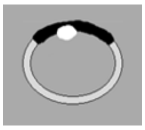

Lymph nodes

☐ No lymph nodes / lymph nodes <5mm

☐ Unsuspected lymph nodes ≥5mm

☐ Suspected lymph nodes ≥5mm

Location of the lymph nodes

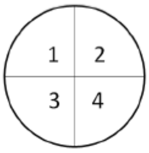

Size of the largest lymph node ….. mm

The lymph node is under / at / above the level of the fibrosis

---

Confidence level tumour response

1. Definitely complete response
2. Probably complete response
3. Maybe (in)complete response
4. Probably incomplete response
5. Definitely incomplete response

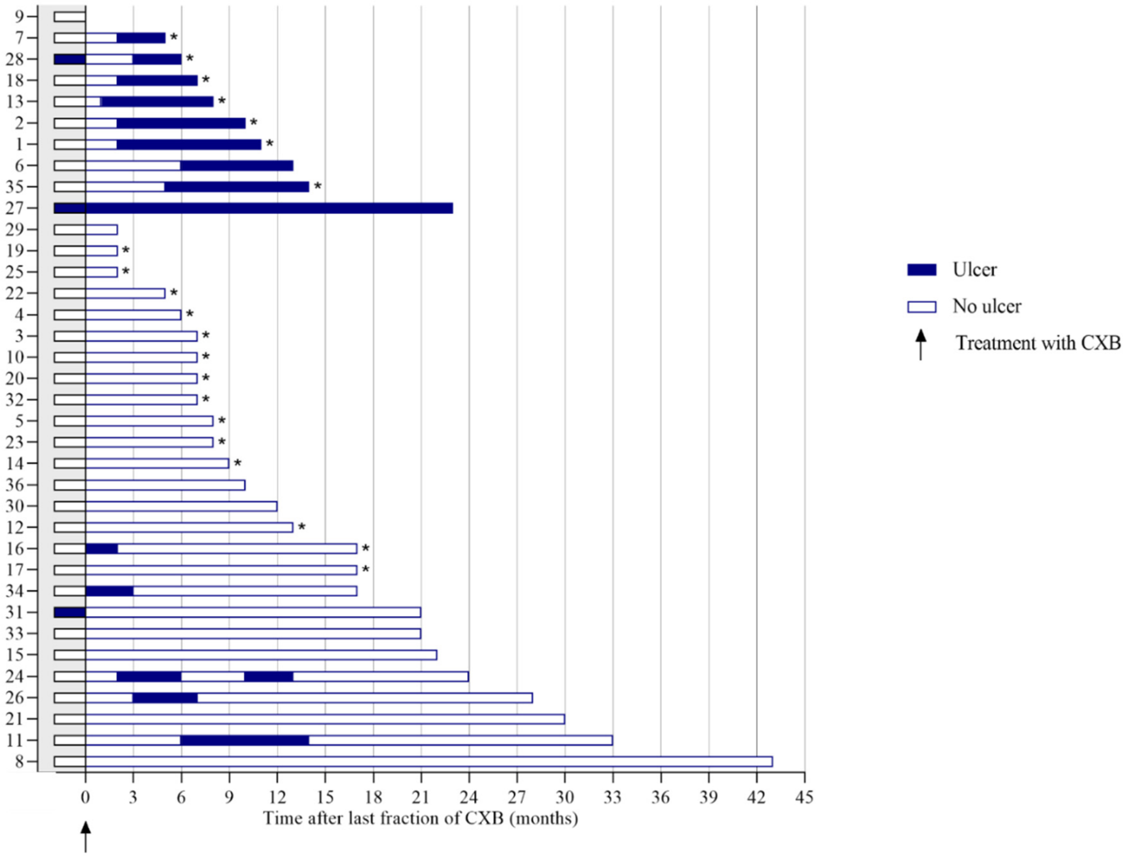

Figure S1. The presence of an ulceration on T2W-MRI prior to and during follow-up after contact x-ray brachytherapy (CXB) per patient. * Patients with histopathological confirmation of residual tumour.

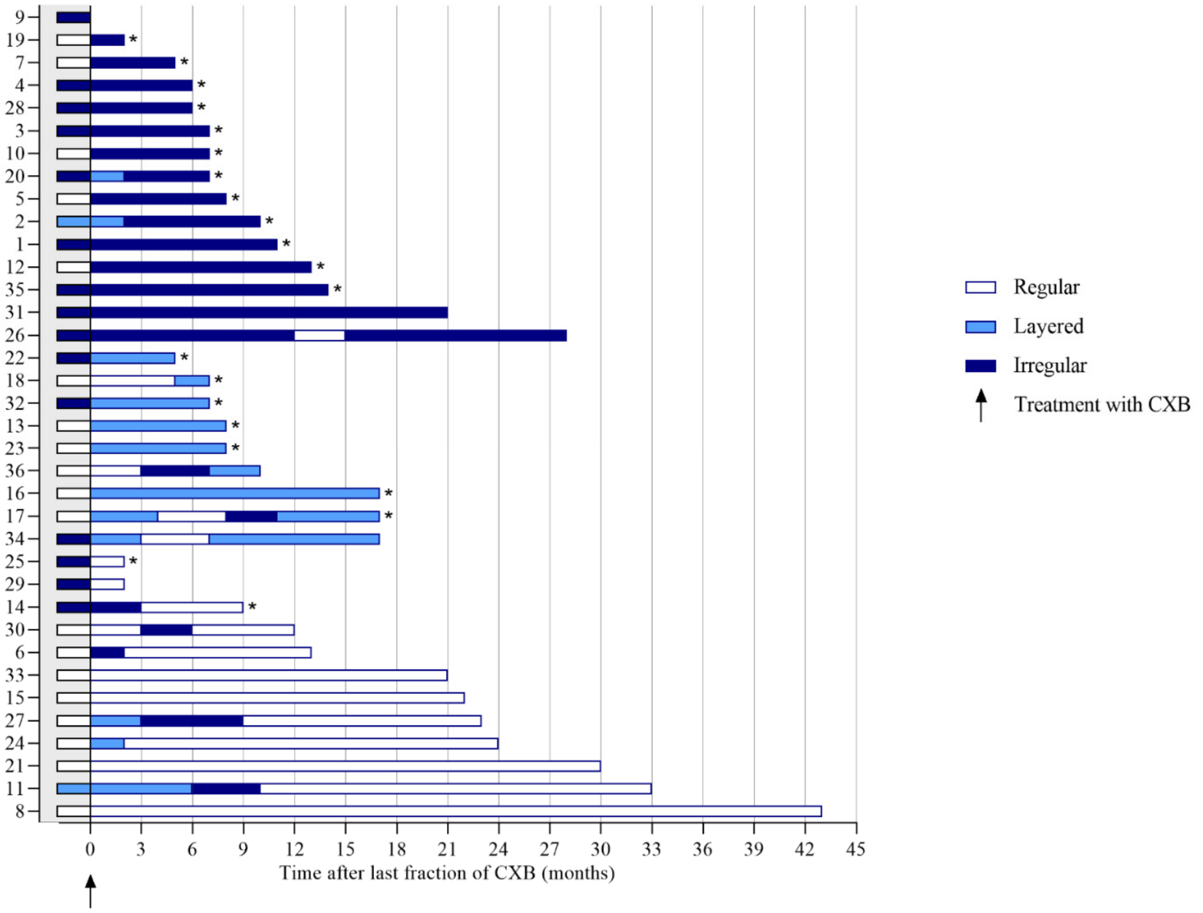

Figure S2. Morphology of the fibrosis on T2W-MRI prior to and during follow-up after contact x-ray brachytherapy (CXB) per patient. * Patients with histopathological confirmation of residual tumour.

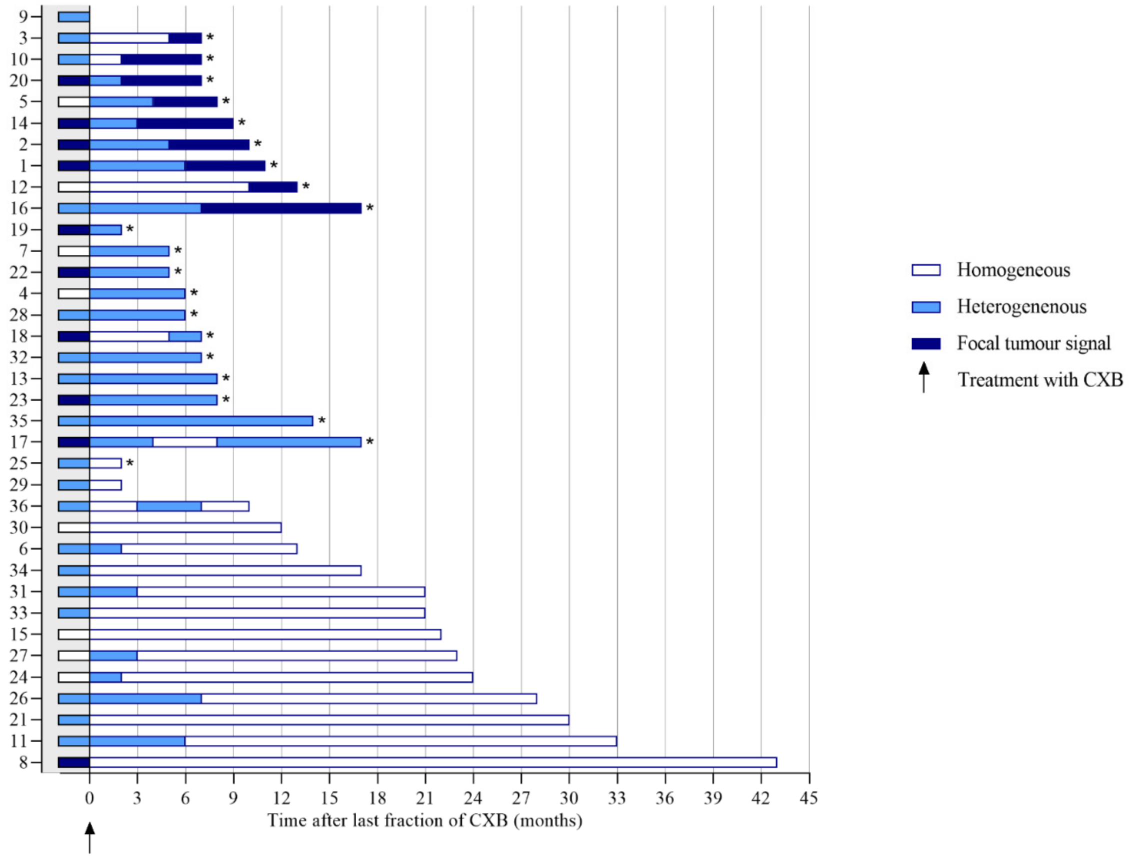

Figure S3: Signal of the fibrosis on T2W-MRI prior to and during follow-up after contact x-ray brachytherapy (CXB) per patient. * Patients with histopathology confirmation of residual tumour.

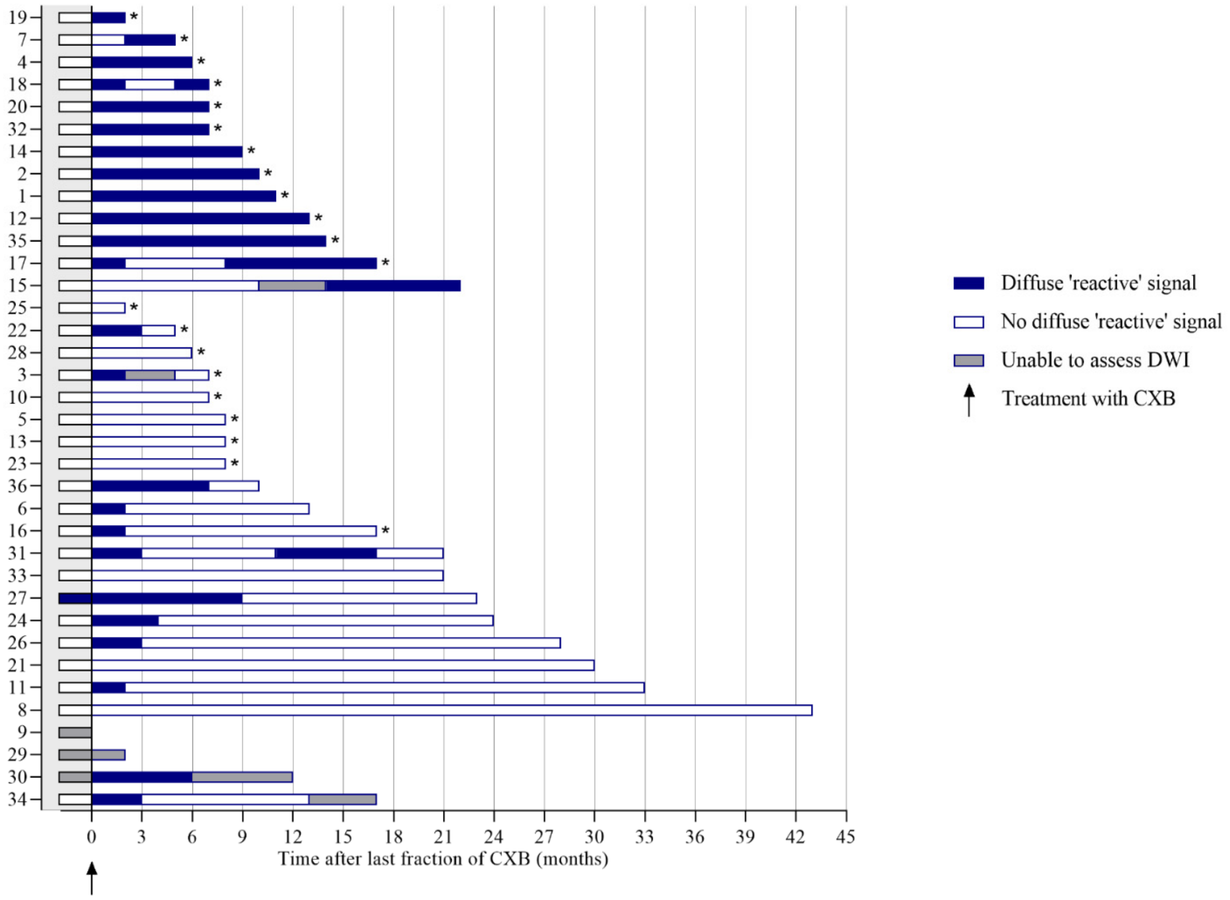

Figure S4: The presence of an "reactive" mucosal signal on DWI prior to and during follow-up after contact x-ray brachytherapy (CXB) per patient. * Patients with histopathology confirmation of residual tumour.
